# A region on the move needs a health workforce trained for it

**DOI:** 10.1016/j.lana.2026.101695

**Published:** 2026-11-05

**Authors:** Báltica Cabieses, Santino Severoni, Patrick Bodenmann

**Affiliations:** a School of Nursing, LKS Faculty of Medicine, The University of Hong Kong, Hong Kong Special Administrative Region of China; b Centre for Global Intercultural Health (CeSGI), Instituto de Ciencias e Innovación en Medicina, Facultad de Medicina Clínica Alemana, Universidad del Desarrollo, Santiago, Chile; c Global Initiative on Health and Migration, World Health Organization, Geneva, Switzerland; d Department of Vulnerabilities and Social Medicine, Unisanté, University of Lausanne, Lausanne, Switzerland; e Chair of Medicine for Vulnerable Populations, Faculty of Biology and Medicine, University of Lausanne, Lausanne, Switzerland

Few regions have seen human mobility change shape as quickly as the Americas. Of the roughly 7.9 million people who have left Venezuela, 6.9 million live elsewhere in Latin America and the Caribbean, 2.8 million of them in Colombia.^1^ Detected crossings of the Darién jungle peaked at 520,085 in 2023, then fell to fewer than 3000 in the first seven months of 2025 as US policy changed; thousands began moving south again, and internal displacement in Haiti reached about 1.3 million.^1^ The health worker who meets these people, whether in a primary care clinic in Cúcuta, an emergency department in Santiago, a shelter in Tapachula, or a community health centre in Chicago, has rarely been trained for the encounter.

The UCL–Lancet Commission on Migration and Health's recent review of progress calls for stronger leadership, accountability, migrant-inclusive services, better data, and attention to the political determinants of health.^2^ These are the right priorities, but each depends on a workforce that needs adequate attention and training. In the Americas, the evidence on that workforce is thin, and what exists is not reassuring.

In the USA, a scoping review of immigrant and refugee health curricula in medical training identified 37 curricula across 21 institutions; 22 were electives, and only four formed part of the core curriculum for all learners.^3^ In Chile, a multiple case study of health sciences programmes in three universities, drawing on 179 students, teachers, and programme directors, found that migrant health was taught largely implicitly, through bioethics and legal content, and that curricula were not aligned with the country's national health policy for international migrants.^4^ Canada has produced a careful undergraduate framework for refugee and migrant health,^5^ but it remains a proposal for schools to adopt rather than a standard they must meet. No regional inventory exists of what schools of medicine, nursing, midwifery, and public health in Latin America and the Caribbean teach about migration, which is, in itself, a finding.

The foundations for reform exist. WHO's global competency standards for health workers, with their accompanying curriculum and knowledge guides, were written to inform precisely this kind of reform.^6^ Yet the region does not need to import frameworks; it has its own to contribute.

Latin American social medicine and critical epidemiology treat health as socially determined, produced by how societies organise work, territory, and power rather than by individual risk factors alone.^7^ Ecosocial theory shows how exclusion, racism, and precarity become embodied biology,^8^ and syndemic thinking explains how structural violence clusters disease and distress among the most exposed.^9^ Intersectionality shows how migratory status compounds gender, race, ethnicity, Indigeneity, and class. A decolonial lens asks whose knowledge counts in health institutions still shaped by the coloniality of power,^10^ while buen vivir, diálogo de saberes, and interculturality, rooted in Andean and other Indigenous thought, frame wellbeing as collective and tied to territory, and care as an exchange between knowledge systems rather than an act of translation. These traditions also widen the gaze from movement to immobility: people stranded in transit, trapped by violence or climate, or held in long waits for legal status, whose health risks are no less real for staying put. Table 1 maps these traditions, alongside the WHO and Canadian frameworks, onto content schools could adopt.

**Table 1:** Migration and health competencies mapped to curricular content for the Americas.

WHO domain (competency standards)	Emphasis in Gruner and colleagues	Contributions from traditions of the Americas	Suggested content for schools in the Americas
People-centredness (standards 1–2)	Social determinants of health; barriers to access; advocacy	Social determination of health and critical epidemiology; social embodiment; intersectionality	Epidemiology of mixed, circular, and return migration; legal status and regularisation as determinants of health; how exclusion becomes embodied across intersecting identities; participatory work with migrant communities
Communication (standards 3–4)	Cross-cultural communication; interpreter use; health literacy	Interculturality; diálogo de saberes	Intercultural mediation; working with interpreters in Haitian Creole, Portuguese, and Indigenous languages; dialogue between biomedical, Indigenous, and Afro-descendant knowledge systems; risk communication along transit routes
Collaboration (standards 5–6)	Systems approaches; interprofessional and community partnership	Buen vivir; collective and territorial approaches to wellbeing	Cross-border continuity of care; community and territorial partnerships; surge preparedness in border and transit areas, including climate-related displacement; recognition of migrant health workers' qualifications
Evidence-informed practice (standard 7)	Global health epidemiology; pitfalls in providing care	Critical epidemiology; syndemic vulnerability; forms of immobility	Migration data systems and their limits; disaggregation by migratory status; syndemic analysis along migration routes; health of people stranded, trapped, or waiting for status; evaluation of migrant-inclusive interventions
Personal conduct (standards 8–9)	Reflective and trauma-informed practice; mental health	Structural violence; decolonial lens	Human rights and professional ethics where enforcement reaches health settings; recognising structural violence in the clinical encounter; reflexivity on coloniality and the practitioner's own position; staff wellbeing

Adapted from the WHO global competency standards^6^ and the framework of Gruner and colleagues,^5^ drawing on the traditions discussed in the text.^7^, ^8^, ^9^, ^10^ The third and fourth columns are the authors' proposal and are not stated by either source.

What is taught should also reflect the region's realities. Graduates should understand mixed, circular, and return flows and how health risks accumulate along transit routes; how legal status shapes access to care, as Colombia's temporary protection for around two million Venezuelans has shown^1^; how to work with interpreters and intercultural mediators in Haitian Creole, Portuguese, and Indigenous languages; and how to practice medicine ethically where immigration enforcement reaches into health settings. Curricula should also recognise migrants as part of the workforce, including clinicians whose qualifications too often go unrecognised.

We propose three steps. First, schools of public health, medicine, nursing, and midwifery across the Americas should make migration and health a mandatory curricular domain rather than an elective. Second, accreditation bodies, from national agencies in Latin America to their counterparts in North America, should write these competencies into programme standards, because reforms that rest on individual champions rarely outlast them. Third, regional networks of schools of public health should support a baseline survey of current teaching so that progress can be measured rather than assumed. The region has shown, through large regularisation programmes, that its health systems can adapt quickly to migration at scale. Its health workforce deserves to be prepared with the same urgency.

## Contributors

BC conceived the Comment, wrote the first draft, and led its adaptation to the Americas. SS contributed the framing of the WHO global competency standards and their application to workforce education. PB contributed the curricular and accreditation arguments and the mapping in the table. All authors revised the manuscript critically for important intellectual content, approved the final version, and agree to be accountable for all aspects of the work. BC is the guarantor.

## Declaration of generative AI and AI-assisted technologies in the writing process

During the preparation of this work the authors used Claude (Anthropic) to help proofread the final version of the text. The authors reviewed and edited the content as needed and take full responsibility for the content of the publication.

## Declaration of interests

This Comment is an original, unsolicited work initiated by the authors. It was not commissioned or requested by WHO or any other organisation, and no has asked the authors to write, be named on, or submit it. BC is a Commissioner of the UCL–Lancet Commission on Migration and Health, whose review is discussed here, and directs a WHO/PAHO Collaborating Centre on migration and health at Universidad del Desarrollo; she receives no personal payment for either role. BC holds a research grant from ANID, Chile (FONIS SA25I0155) on intercultural mediation for migrant populations in primary care, paid to her institution. SS is a staff member of WHO and Director of its Global Initiative on Health and Migration, which produced the competency standards cited in reference 6; the views expressed are his own and do not necessarily represent the decisions or policies of WHO. PB co-directs a WHO Collaborating Centre on migration and health at Unisanté, holds the Chair of Medicine for Vulnerable Populations, and is Vice-Dean for Education at the Faculty of Biology and Medicine, University of Lausanne. We declare no other competing interests.
